# Decision-making during training of a Swedish navy command and control team: a quantitative study of workload effects

**DOI:** 10.1007/s10339-024-01242-9

**Published:** 2024-11-13

**Authors:** Marie Hindorf, Denise Bäckström, Carl-Oscar Jonson, Anders Jonsson, Peter Berggren

**Affiliations:** 1 Department of Biomedical and Clinical Sciences, Linköping University, Linköping, Sweden; 2 Swedish Armed Forces, Naval Warfare Centre, Vallgatan 11, 371 31 Karlskrona, Sweden; 3 Center for Disaster Medicine and Traumatology, Linköping, Sweden; 4 Department of Caring Science, University of Borås, Borås, Sweden; 5 Swedish Armed Forces, Centre for Defence Medicine, Göteborg, Sweden; 6 Department of Computer and Information Science, Linköping University, Linköping, Sweden

**Keywords:** Simulations, Workload, Team training, Command and control

## Abstract

The study compared two simulation environments for training of Swedish naval Command and Control teams by using indirect measures, including workload, combat readiness, and situation awareness. The literature explains simulation-based training as providing a safe avenue to practice relevant scenarios. Fidelity, the degree of realism in the simulation, and workload, the equilibrium between demands and assigned tasks, are crucial factors examined in this study of low- and high-fidelity naval simulations. This study was conducted to better understand the effects of various training methods. An experimental design with repeated measures was used with three consecutive escalating parts. The subjective, multidimensional assessment tool, NASA-Task Load Index was used to rate perceived workload. Combat readiness of the ship and mental demand yielded significant results. For combat readiness of the ship, there was a difference between the low and the high-fidelity setting, for the initial part of the scenario *p* = 0.037 and for the second part *p* = 0.028. Mental demand was experienced as higher in the low-fidelity setting, *p* = 0.036. Notably, the simulated internal battle training for onboard command teams in a low-fidelity setting was found to induce a level of stress comparable with that experienced in a high-fidelity setting. The results indicate that low-fidelity training results in a workload not distinguishable from high-fidelity training and has practical implications for increased use of low-fidelity training as part of (naval) command team training programmes.

## Introduction

Warfare and battlespace have evolved dramatically over the past century (Brands 2024; Jayamaha and Matisek 2022). To address the challenges of modern warfare, it is vital for the Armed Forces to perform training for a range of potential scenarios. In the Swedish Armed Forces both large-scale and small-scale exercises are essential for preparing the units. Both types of exercises often involve event simulations to prepare the units for the various situations they might face. Some exercises are minor, involving only a few participants, while others are extensive and sometimes involve international collaboration. These larger exercises focus on enhancing cooperation with foreign military forces and improving integration among different units within the Swedish Armed Forces (Swedish Armed Forces 2024a, b).

### Navy background

The Swedish Navy performs large-scale simulations on a regular basis, both nationally and internationally. These are planned in an annual maritime training and exercise programme. Minor simulations are sometimes carried out at the flotilla level, onboard ships, or before large-scale simulations. Extensive/large scale maritime exercises often include participation from other countries, such as the annual naval exercise Baltic Operations (Baltops). This year marked Sweden's first participation in Baltops as a NATO ally (Swedish Armed Forces 2024a, b).

When Swedish naval ships are exposed to weapon-induced damage or other unexpected events, the so-called internal battle or internal combat, also known as damage control (henceforth called internal battle), has to function onboard to be able to maintain combat readiness (Swedish Armed Forces 2018). The internal battle includes the ability to prioritize actions inside the ship’s hull, for example, the ability to handle fires, damage control or casualty care. Most internal battle education and training is conducted at the Swedish Naval Warfare Centre in the south of Sweden where naval officers, sailors and others are trained.

In summary, Sweden has a long history of maritime education and training (MET), but the specifics of how this training should be conducted/designed to support most effective team training is lacking.

### Performance framework

In the 1990s and beyond, the US Naval Air Warfare Center Training System Division (NAWCTSD) explored performance and workload impacts in naval Command and Control (C^2^) teams. The Tactical Decision Making Under Stress (TADMUS) program resulted in innovative interventions like Team Dimensional Training (TDT) (Johnston et al. 1998; Smith-Jentsch et al. 1998a, b). The concept of Cockpit (later: Crew) Resource Management (CRM) was also introduced in the 1990s (Bowers et al. 2000).

The performance of naval command and control (C^2^) teams involves decision-making by subordinate units/individuals and reporting to the higher level. Assessing the quality of this performance is quite challenging. Naval C^2^ team training can be conceptualized within the framework of simulation-based training (SBT). SBT have been used previously in MET (Wiig et al. 2023), and existing research highlights the prevalent use of simulations in military contexts, emphasizing the importance of training in a nonviolent environment before exposure to conflicts (Moses et al. 2001; Salas et al. 2008). SBT finds application in both individual and team training (Moroney and Lilienthal 2009; Paige et al. 2014) extending to medical education (Hegland et al. 2017; Marker et al. 2019; Tosterud et al. 2013) and simulations related to crisis management (Saetren et al. 2024). Notably, SBT is used in scenarios involving the observation of team performance among escalating and unforeseen events (Bergström et al. 2010). For instance, it is utilized in evaluating the effectiveness of elite firefighting teams during simulated exercises (Vidal and Roberts 2014). Naval C^2^ teams face similar issues as crisis management teams such as time pressure, allocation of resources or objective conflicts. There are thus different types of performance framework but there are also different fidelities to consider.

### Fidelity

When practising complex tasks, SBT is a safe method to use (Salas et al. 2008; Lateef 2020) and simulations should be as close to reality as possible (Dieckman et al. 2007). The degree of realism in SBT is referred to as fidelity and is created through the simulation equipment, setting, and scenario (Choi and Wong 2019). Fidelity is described as the degree of similarity between the simulated and the operational situation (Singer and Hayes 1989). Kim et al. (2016) discuss three types of simulation settings and the levels of ability reached by the settings; low-fidelity simulation (builds knowledge), mid-fidelity simulation (builds competence) and high-fidelity simulation (builds performance). In this study, two levels of settings were used; low- and high-fidelity. There are different ways to look at fidelity and according to Rashid and Gianduzzo (2016), there are three primary types of fidelity: physical, conceptual, and psychological. Fidelity is also about how precise the simulation is (Adamson 2015; Rashid and Gianduzzo 2016), and trust the experience of the simulation (Cook et al. 2013). Low-fidelity simulations are described as simple (Banks 2010), and can be based on a paper or computer task, or involve the use of mannequins with modest interactions, for example, basic cardiopulmonary resuscitation training.

Another example of low-fidelity simulation is the Crew Resource Management (CRM) training which has developed over the years and is part of education and training in aviation (Tsz-Kin Chann and Li, 2022), in training of trauma personell (Ashcroft et al. 2021) and in the naval context where a suggestion of changing CRM into bridge resource management (BRM) instead which may be decisive for the success of naval maritime operations (Campaniço Cavaleiro et al. 2020). Wahl et al. (2018) emphazise that the CRM training in the maritime industry should be customized for current crews, with a learning environment as close to reality as possible.

The disadvantage of low-fidelity simulation is the low level of realism, and the fact that participants are not exposed to a real-life setting. High-fidelity simulations are described as complex and close to reality (Banks 2010) with a high level of realism that brings the participant into an authentic work environment; for example, in maritime simulators (Waldenström, 2012; Tusher et al. 2023) or in a helicopter fuselage submerged in a swimming pool for evacuation training (Brooks 1997; Vicente-Rodríguez et al. 2020, 2024). Research indicates that high-fidelity simulations are not necessarily considered to be more stressful (Finan et al. 2012) and may result in overconfidence (Masiello and Mattsson 2017; Massoth et al. 2019). The perception of higher levels of fidelity as being superior to the lower levels is common among both participants and instructors, but there is no clear evidence to support this statement (Fragapane et al. 2018; Kim et al. 2016; Norman et al. 2012). High-fidelity simulations also require extensive resources; for example, finance, time, and personnel (Fragapane et al. 2018; Massoth et al. 2019; Norman et al. 2012). How well SBT mimics real working conditions is often expressed in terms of simulation fidelity; the degree of realism in the simulated physical, psychological and conceptual environments.

### Workload, combat readiness and situational awareness

The evaluation of the factors that influence the performance of naval C^2^ teams, such as workload, combat readiness, and situational awareness (SA) is essential (Holthausen et al 2019; Michailovs et al 2023). These factors can significantly impact performance and thereby play a crucial role in the unit’s capacity to effectively address assigned tasks. Workload can be characterized through various perspectives, such as the ratio of the tasks assigned to an individual compared to the actual completed work and the subjective perception of workload (Jex 1998) or the equilibrium between demands and tasks (Leedal and Smith 2005). In exploring the correlation between workload and performance, existing research indicates that increased workload tends to correspond with diminished performance (Brookhuis and De Ward 2010; Young et al. 2015). Mental workload is delineated as the disparity between task demands and the resources available (O’Donnell and Eggemeier 1986). Workload can be measured from various aspects; one validated tool is the National Aeronautics and Space Administration-Task Load Index (NASA-TLX) instrument. NASA-TLX is a subjective, multidimensional assessment tool used for rating perceived workload with the following six subscales: mental demand, physical demand, temporal demand, performance, effort, and frustration (Hart 2006). The perceived workload can be assessed as high or low in relation to a global set of NASA-TLX scores from a meta-review by Grier (2015). NASA-TLX has been used in crisis management research (Hoonakker et al. 2011; Huggins and Claudio 2018; Levin et al. 2006; Nygren 1991; Saleem et al. 2007; Szalma et al. 2004; Weinger et al. 2000; Yurko et al. 2010). A Swedish version of the NASA-TLX form was translated by Prytz et al. (2016) for use in a multi-agency exercise to compare workload between command teams, similar to the Swedish Naval C^2^ team. Due to the significant need for MET as well as the challenges in understanding differences in fidelity, we choose to use the NASA-TLX as a method in this study.

Readiness within MET and the military is characterized by the military’s capability to actively engage in combat and execute assigned missions and tasks within diverse timeframes, ranging from minutes to months (Herrera 2020; Junor 2017). Combat readiness extends to different levels, addressing both individual and collective states of readiness (Kruys 2001). Individual combat readiness encompasses psychological and equipment-related aspects, from mental preparedness for combat to having personal protective equipment in order (Hindorf et al. 2018, 2020; Schraagen and Post 2014). The holistic combat readiness of a ship encompasses factors such as crew fatigue, training protocols, and available resources (Comperatore et al 2005; Moundalexis et al 2011; Lützhöft et al 2011). Notably, research on naval combat readiness specific to naval C^2^ teams is either outdated or predominantly focused on team effectiveness and performance (Goodwin et al. 2018; Kaempf et al. 1996). Explicitly, in this study combat readiness is seen as a precursor to performance.

When examining situation awareness (SA), the term is viewed as “a state of knowledge”, and to achieve that knowledge situational assessment is viewed as “processes” (Endsley 1995a). SA is also described as “the perception of environmental elements and events with respect to time or space, the comprehension of their meaning, and the projection of their future status” (Endsley 1995b, p. 36). Endsley (2011) provided a theoretical framework of SA and included three levels of SA: perception, comprehension, and projection. SA can be divided into different parts, and, for example, has been referred to as the actual awareness of the situation (Endsley 2011; Lundberg 2015). SA is sometimes mixed up with the term situational understanding, which is a broader view of the situation when interpretation and comprehension of SA are done (Endsley 2021). In this study, SA is referred to as handling the workload in both tedious and unexpected events.

In summary, naval training and the level of realism (fidelity) may have an impact on workload, combat readiness and SA.

### Objectives

There is a need for a deeper understanding of how simulation fidelity affects performance in naval training, specifically for C^2^ teams. This explorative study was conducted to examine the effects on the simulation environment in both a low-fidelity and a high-fidelity naval context using indirect measures. The study was performed to gain basic data and see what was discovered to increase our knowledge in the area. For this reason, we chose to do an explorative study. To our knowledge, comparison of low- and high-fidelity simulation settings during SBT for Swedish naval C^2^ teams has not been measured this way before.

The objective was to compare two simulation environments for training of naval C^2^ teams using indirect measures: workload, combat readiness and SA.

### Research questions

- RQ 1: Do low- versus high-fidelity simulation settings differ in the workload experienced using NASA-TLX?
- RQ 2: Do low- versus high-fidelity simulation settings differ regarding perceived combat readiness (subjective performance)?
- RQ 3: Do low- versus high-fidelity simulation settings produce different results regarding SA?

## Materials and methods

### Design

**Fig. 1:**
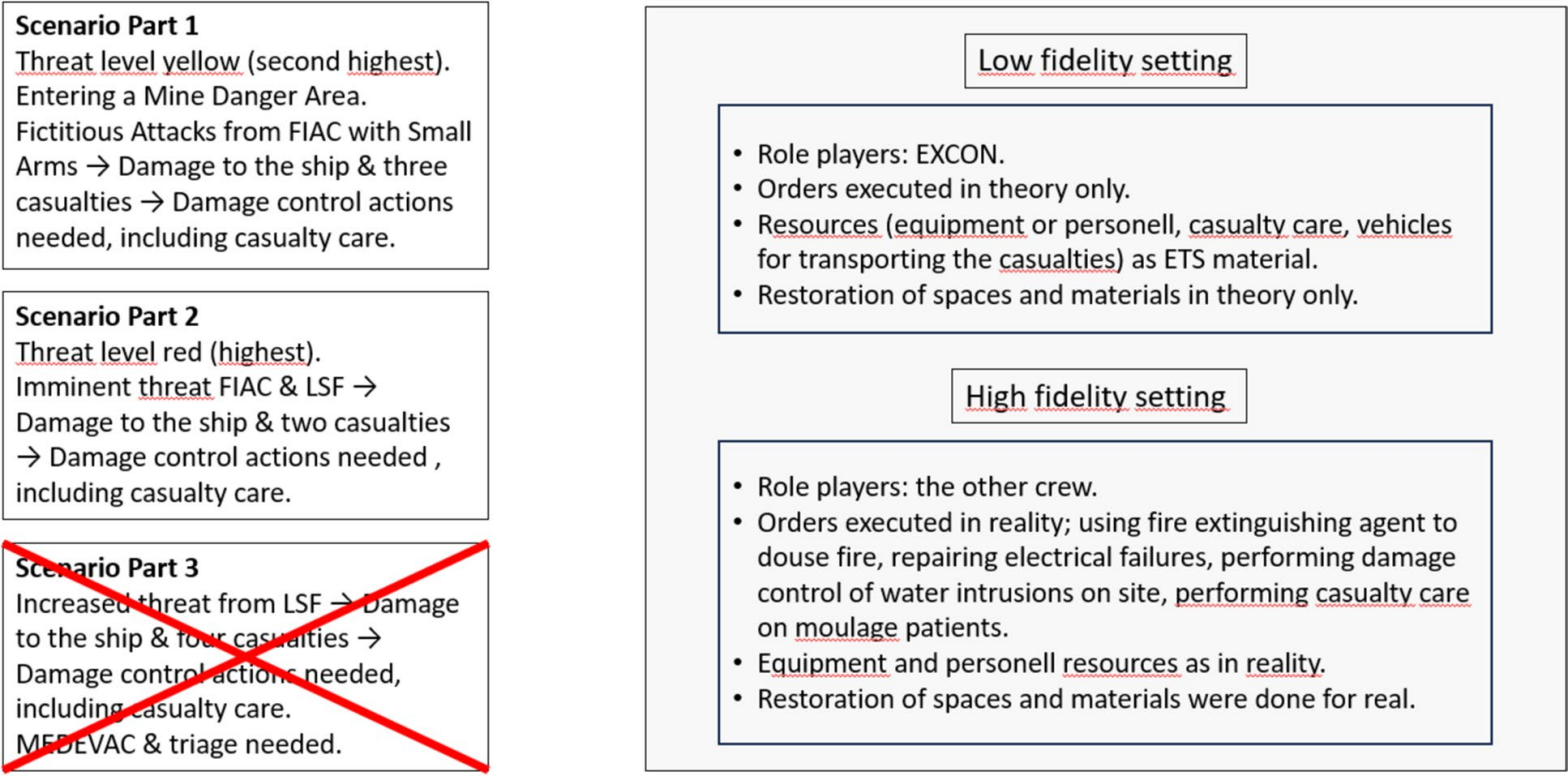
The scenario and differences between actions made in the low- and high-fidelity setting

An experimental design with repeated measures was used in the scenario. The independent variables were the three consecutive escalating parts as described in the scenario section; the context included two levels: a low-fidelity simulation setting (Emergo Train System [ETS] with a Navy module) and a high-fidelity simulation setting (the Vulcanus II training facility for fire protection and related ship training). The dependentvariables were NASA-TLX, combat readiness (of the ship, physical and psychological), and SA. Participants’ ratings for personal psychological combat readiness on a strategic level, personal physical combat readiness, SA, and overall combat readiness of the ship were included. The six dimensions of NASA-TLX; mental demand, frustration, effort, temporal demand, performance, and physical demand (Hart 2006) were assessed, and a combined measure of the overall NASA-TLX workload was computed. NASA-TLX was collected during the C^2^ team’s simulation based inner battle training in the two settings. In Fig. 1 below, an overview of the inner battle training for the C^2^ team is presented, including the scenario and differences in actions taken in the two different settings.

### Participants

#### Command and control team

In the escalating scenario, the C^2^ team had the task of conducting logistics support in the archipelago. The selection process for being part of a C^2^ team onboard is rigorous, requiring extensive experience in the Swedish Armed Forces, application of training, high recommendations, and a suitability assessment. This formalization is essential for specific positions onboard, such as being part of the C^2^ team. Detailed selection criteria regarding the command team cannot be specified due to operational confidentiality. Given that decision-making originates from the chain of command onboard naval warships, the participants selected for this study align with its objective, encompassing the entire naval C^2^ team on board a vessel. Ten participants were selected and recruited from the naval C^2^ team on HswMS Carlskrona in the Swedish Royal Navy: the Commander (CO); Second in Command/Executive Officer (XO) who was also the Internal Battle Commander (IBC); Weapon Engineering Officer Electronics; Weapon Engineering Officer Weapons; Machine Engineering Officer who was also the Damage Control Officer who leads and allocates resources within damage control, which include recoverability activities in a ship before, during, and after events/incidents; Machine Engineer 1; Machine Engineer 3; Medical Officer who was also a registered nurse, and two incident plotters (comms personnel). The crewmembers who were not involved in the naval C^2^ team (i.e., non-participants in the present study) are described as the other crew.

### Simulation environments

#### ETS

The ETS material included magnetic symbols on whiteboards to simulate internal battle processes. Maps, tables, pictures, and ship models were arranged to replicate the training facility Vulcanus I.

The ETS simulation system has been used previously for command crews as part of research and evaluation of ETS projects (Hermelin et al. 2020; Nilsson et al. 2015; Rybing 2016, 2018). ETS was used in this study to facilitate the tabletop exercise (TTX), and to test the actions taken by the Swedish naval C^2^ team onboard. TTX is an SBT aiming to discuss details in a simulated scenario and can be simple or complex with an advanced escalating scenario to create conceptual understanding or identify areas for improvement (Prytz et al. 2017). Casualties suitable for this scenario were pre-selected from the ETS victim bank and were utilized throughout the SBT. The Centre for Disaster Medicine and Traumatology in Linköping, Sweden, has developed a naval module of ETS in collaboration with the Swedish Naval Warfare Centre, which includes the Navy’s specific organization, positions, roles, tasks, and equipment. The Navy ETS simulation was conducted indoors, and different classrooms/conference rooms were used to represent spaces for the naval C^2^ team to accomplish their duties as they would onboard, for example, at the mine deck. The naval C^2^ team could easily take part in a command huddle or gathering to exchange information. The exercise control team (EXCON) from the Naval Warfare Centre were used as role players in the SBT, and they provided information about the consecutive escalating parts of the scenario. The C^2^ team issued orders to the simulated crew, and the EXCON team received and executed the orders given. The same kind of communication devices were used during the ETS scenario and the Vulcanus II.

**Fig. 2:**
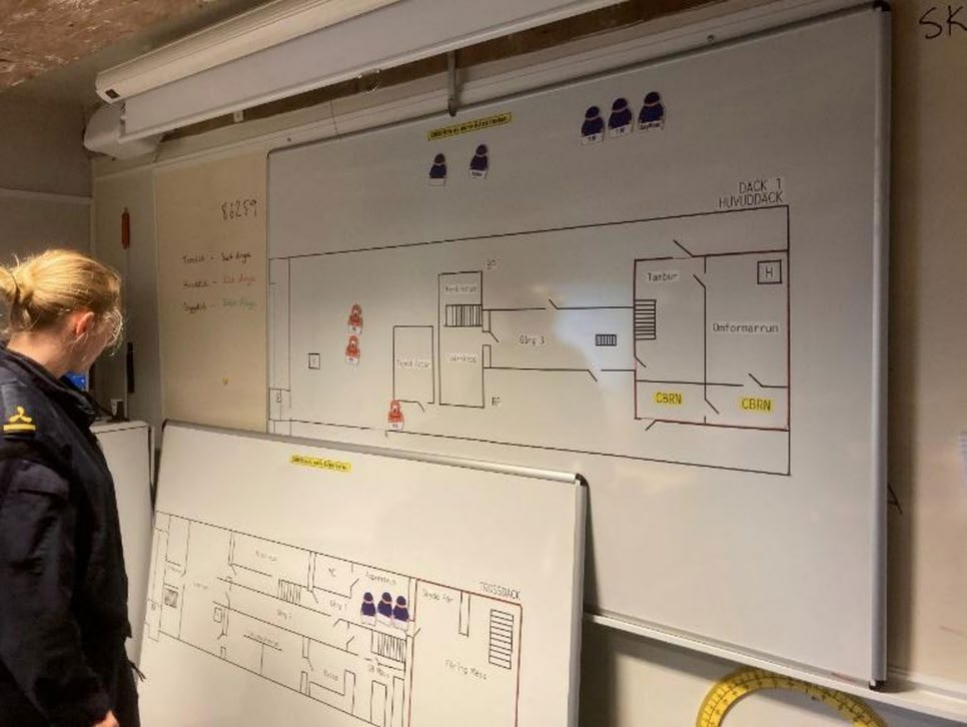
Example of an initial line-up of ETS Navy with ship model, deck and spaces arranged in the same way as onboard Vulcanus II. Photo: Peter Berggren, Center for Disaster Medicine and Traumatology in Linköping

**Fig. 3:**
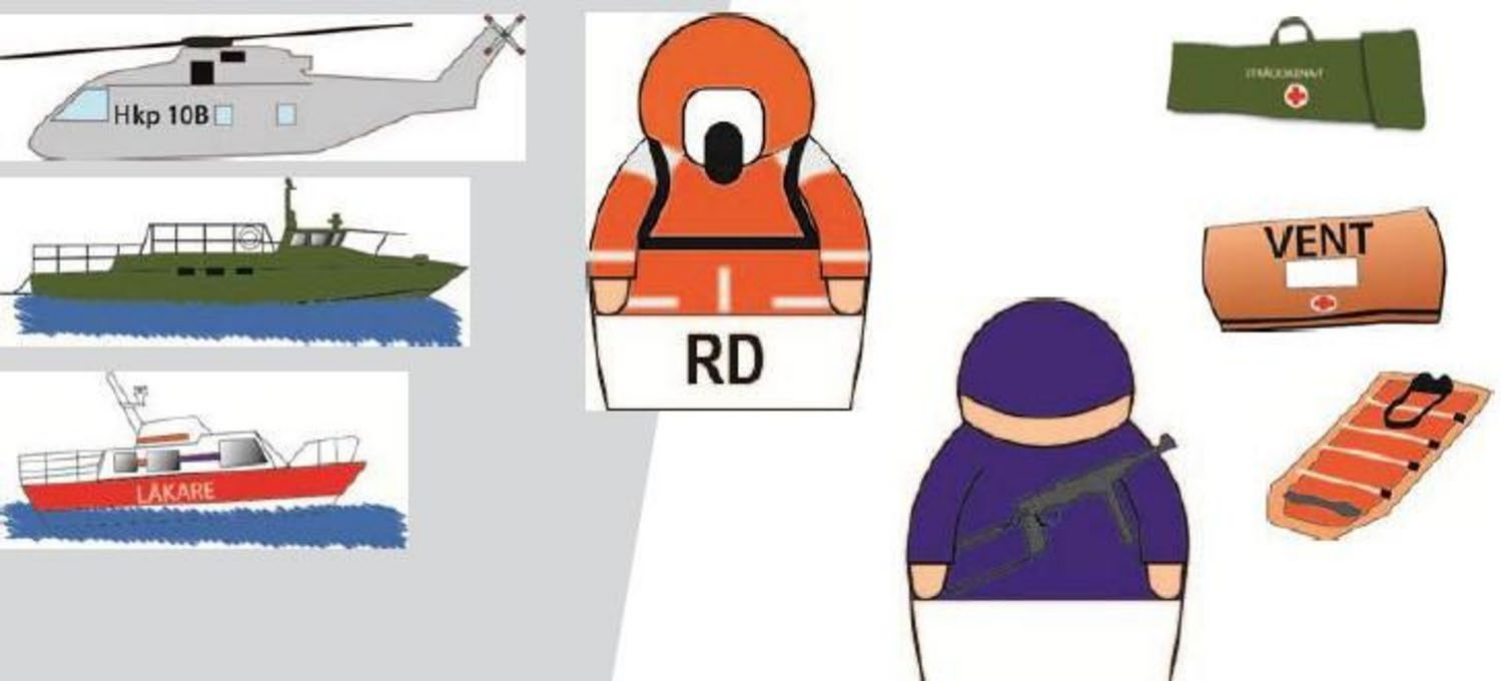
Example of resources used in ETS Navy. Photo: Center for Disaster Medicine and Traumatology in Linköping

**Fig. 4:**
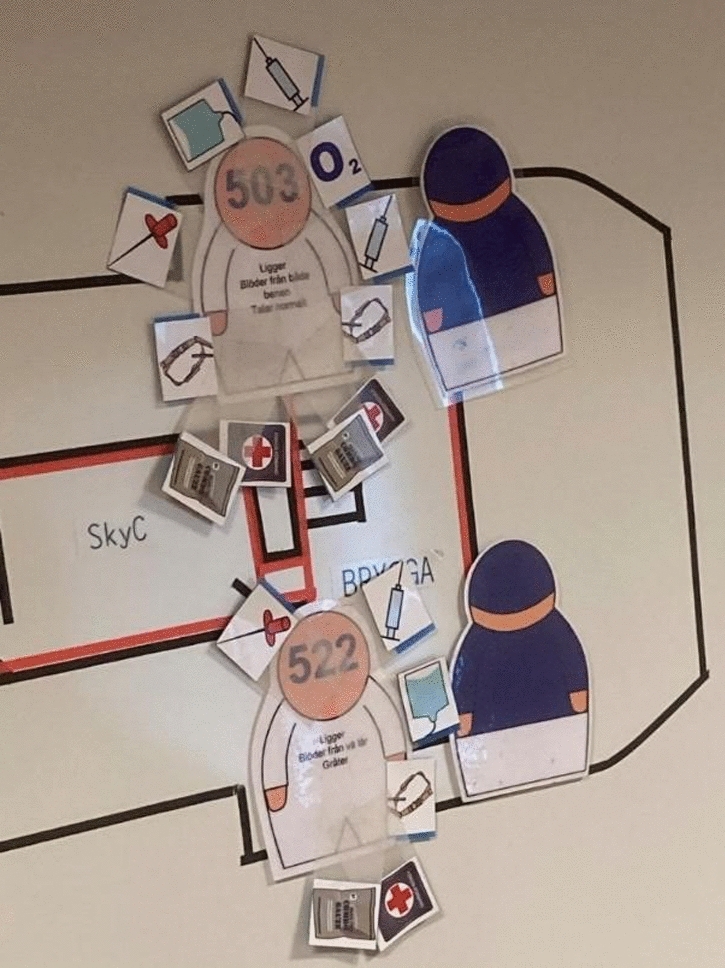
Example of ETS Navy Magnetic symbols (casualties and crew) and actions taken as sticky notes on the simulated casualties. Photo: Peter Berggren, Center for Disaster Medicine and Traumatology in Linköping

The low-fidelity scenario was set up to enable the participants to conduct work as realistically as possibly (Fig. 2), using ETS material and resources such as vehicles for transporting the casualties from the vessel, equipment or personell (Fig. 3). In Fig. 4, examples of how actions was made when performing casualty care in theory.

#### Vulcanus II

The training facility platform for fire protection and related ship training, Vulcanus II (Swedish Armed Forces 2012), is located at the Swedish Naval Warfare Centre and the Sea Safety Training School. The meaning of “related ship training” is for example MET in basic safety or internal battle organizations and techniques. The platform is constructed from containers to imitate a ship with spaces that correspond to the spaces onboard a ship, such as the engine room or the galley. When using Vulcanus II, there are often many instructors involved with additional temporary reinforcements in certain areas depending on the level of the participants as well as the aim of the simulations.

**Fig. 5:**
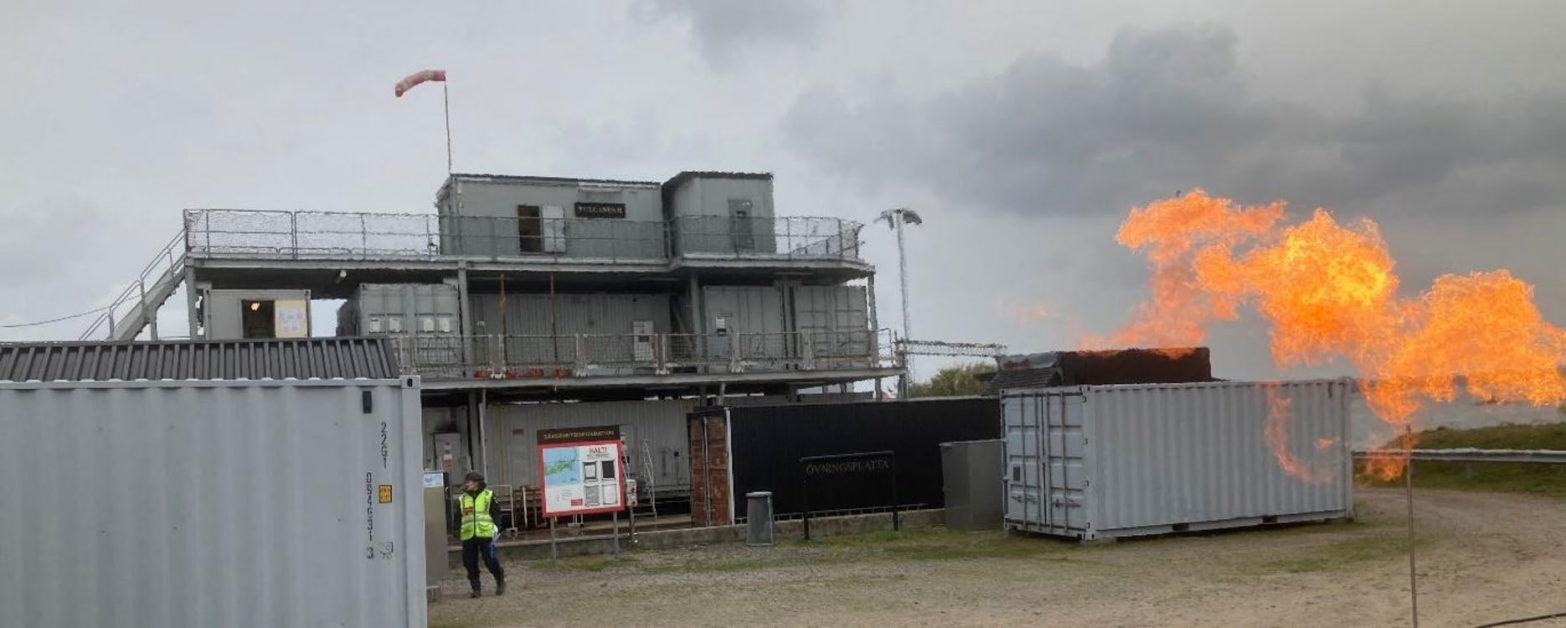
Vulcanus II construction. Photo: Peter Berggren, Center for Disaster Medicine and Traumatology in Linköping

**Fig. 6:**
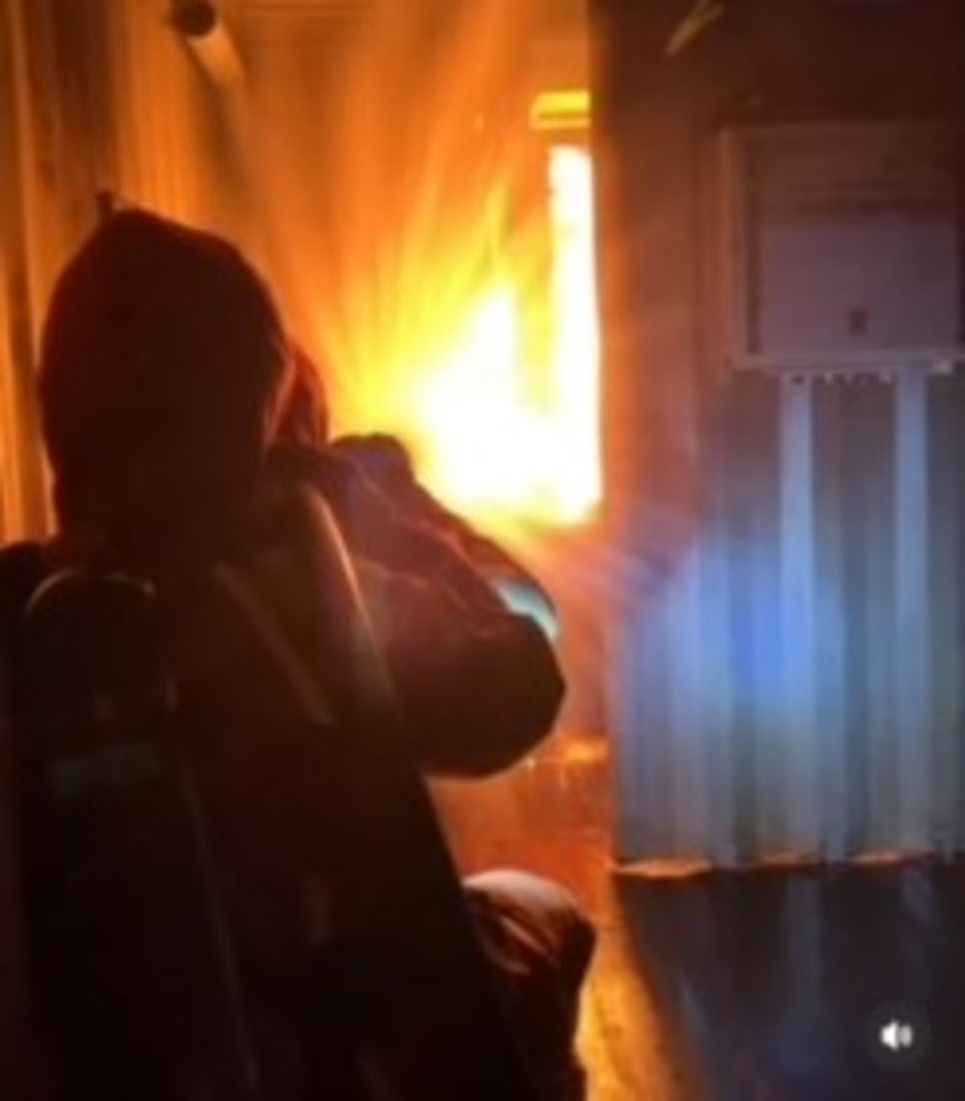
Crew executing set orders for firefighting. Photo: Swedish Naval Warfare Centre, Swedish Navy Damage Control Training

**Fig. 7:**
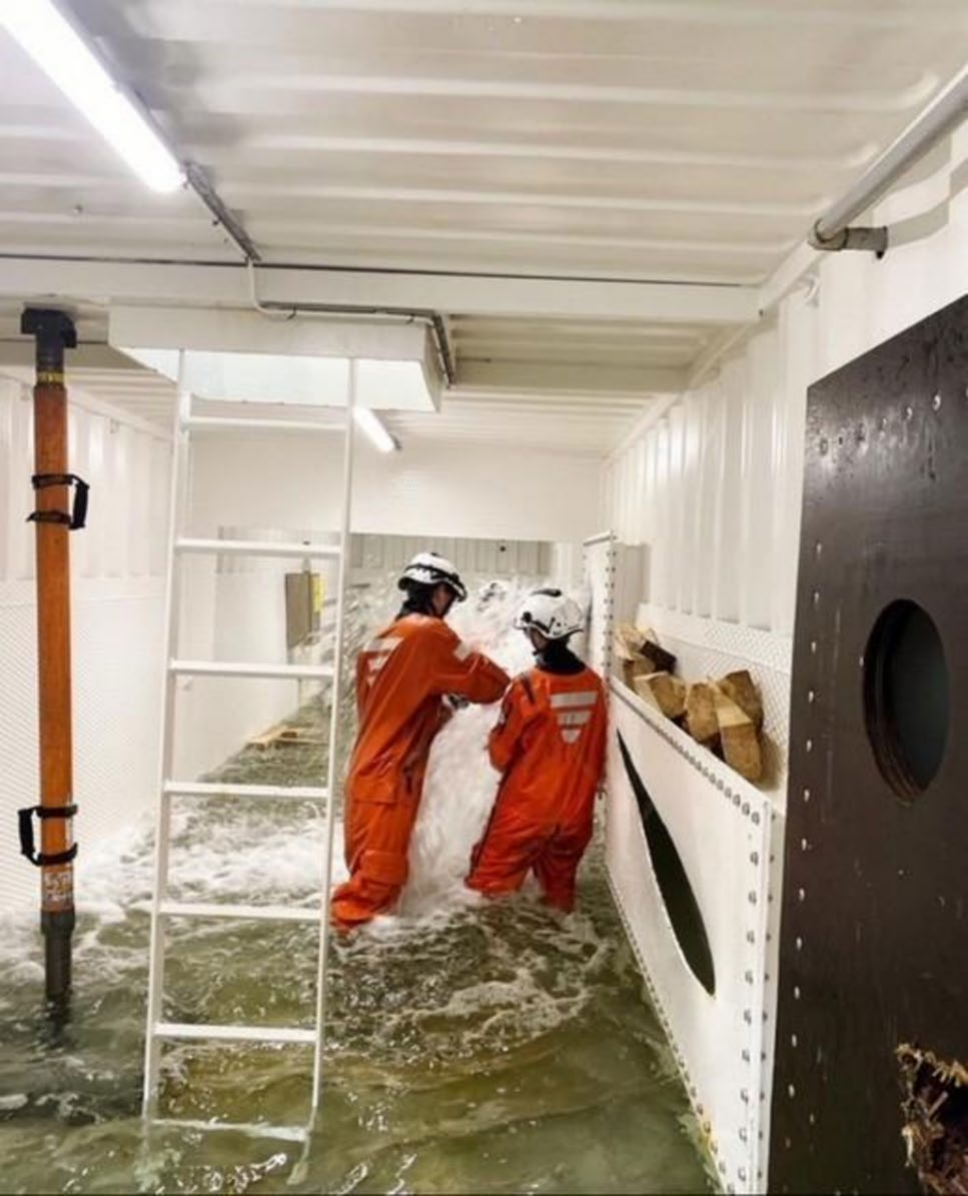
Crew executing set orders for damage control of water intrusion. Photo: Swedish Naval Warfare Centre, Swedish Navy Damage Control Training

**Fig. 8:**
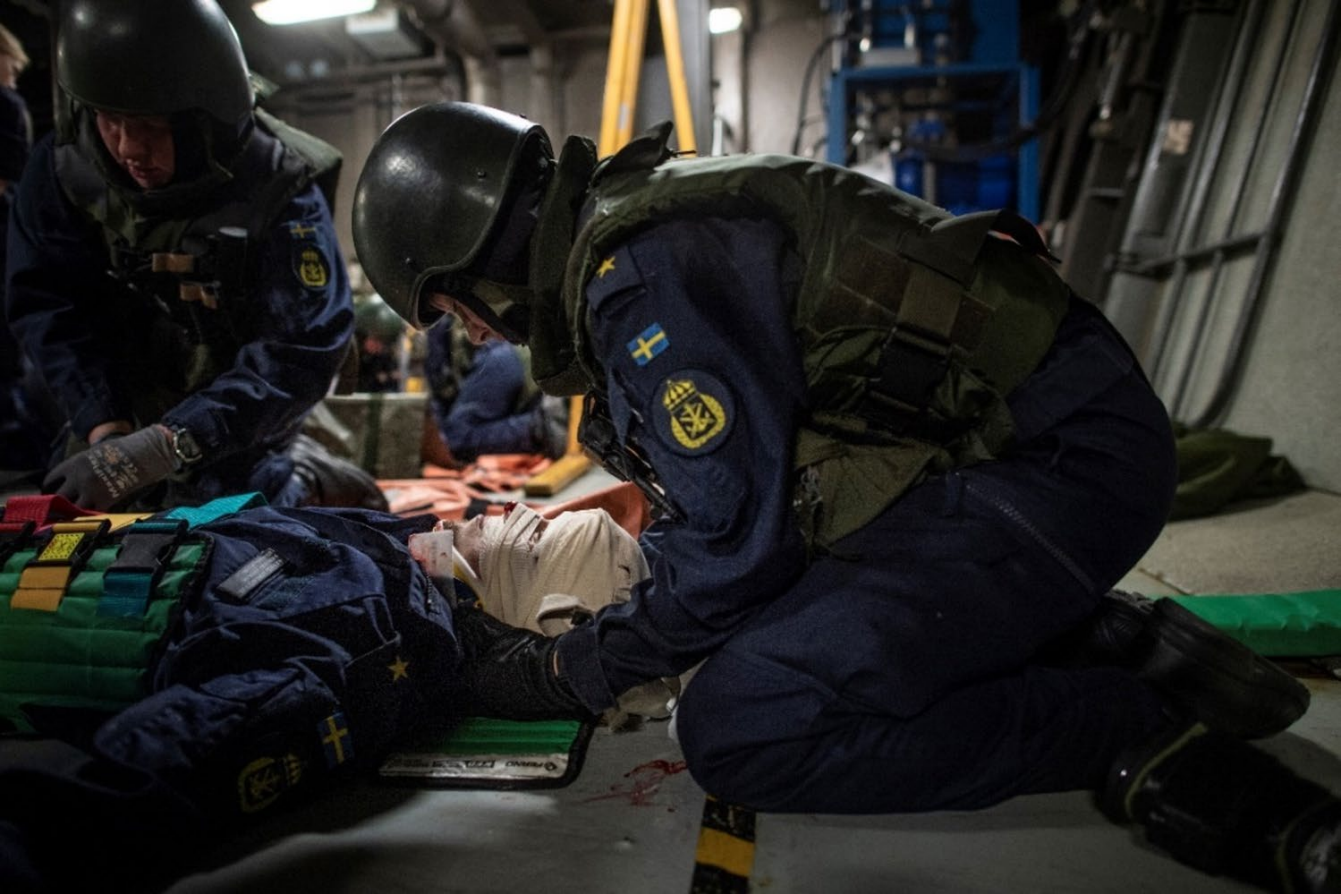
Crew executing set orders for casualty care. Photo: Swedish Naval Warfare Centre, Swedish Navy Damage Control Training

In this study, the other crew was used as role players in the SBT. The simulations onboard Vulcanus II were accomplished either inside the “ship’s” hull or outside on deck, and the distance between the naval C^2^ team and the other crew increased. The training facility enabled the other crew to perform firefighting simulations, hull damage control or simulated casualty care onboard. The other crew enforced the prioritized actions and reported back to the naval C^2^ team. The XO/IBC had to assess the total damage situation by a moving management methodology, which means constant information retrieval onboard the ship. Figure 5, shows the training facility platform Vulcanus II. Figures 6, 7 and 8 shows the actions made by the crew when executing set orders at the training facility.

### The scenario

In October 2020, an SBT was conducted with the naval C^2^ team and part of the crew on a vessel in the Swedish Navy, HSwMS Carlskrona, with the aim of training the ship’s C^2^ team in internal battle simulations onboard. In this study, the ETS Naval module and the training facility Vulcanus II were used.

The scenario was based on simulated internal battle training for a naval C^2^ team that was required to accomplish redistribution of personnel and material, an overview of the scenario is presented in Fig. 1. The same scenario was used twice, in the morning with the naval C^2^ team and in the afternoon in collaboration with the other crew. At the start of the scenario, the participants went to their battle stations for threat level yellow (second highest). Three consecutive escalating parts were included in the scenario. The complexity of the developed scenarios progressively increased with each part of the scenario. Part 1 involved entering a mine danger area with tactical movements suitable for both the ship and the conditions in the area. The focus was on how the C^2^ team handled the security for the personnel on deck. The scenario then escalated with fictitious attacks from a Fast Inshore Attack Craft (FIAC) with small arms that resulted in damage to the ship and three casualties onboard. At the same time, electrical failures and fires needed to be taken care of and both spaces and materials onboard had to be restored. Again, small arms were fired towards the ship, and this time the cooling pipes on the port side broke, which needed damage control. Part 2 involved imminent threat from the sea and air from a FIAC and a Low Slow Flyer (LSF). The fictitious attacks were intensified with increasing threat levels, culminating with level red (highest). Then a malfunctioning fire detector caused a fire alarm from inside the ship’s hull. At the same time, fires were raging in the machine room. Two crew members were injured during the attack. Part 3 involved an escalating scenario with increased threat from an LSF. Damage control, firefighting, and casualty care of the wounded were needed. Another four individuals from the crew were injured. To conclude the scenario, medical evacuation (MEDEVAC) was made possible with a helicopter, and triage was needed for nine casualties.

### Material

Supplementing material in Swedish, such as questionnaires and other information, are available upon request (Berggren et al. 2020). Workload (subscales and total score) was measured by NASA-TLX with a score from 0 to 100, where 0 was low, simple, not working, no effort, calm and 100 was high, complex, perfect, max capacity, demanding/stressful, etc. (Hart 2006). Physical value, mental value, SA and combat value of the ship were measured in the same way.

### Procedure

When the Swedish naval C^2^ team and the other crew arrived at the Swedish Sea Safety Training school in the morning, the event began after a short presentation of the EXCON, the instructors and the schedule for the day. The other crew prepared themselves and the training facility for the SBT scheduled for after lunch, and the naval C^2^ team began with the simulated internal battle scenario, partly visualized by Navy ETS. The TTX was conducted using magnetic symbols placed on whiteboards to illustrate the different events onboard. Other items such as charts and plots of the general arrangements of the Vulcanus II were used to facilitate the simulation as well as checklists for war briefs and the commander’s aim. The aim of war briefs is to inform the entire crew of the upcoming task and its cause; they must be short and precise with orders from the upper level of the chain of command and must be performed before the new task begins or on leaving port (Swedish Armed Forces 2020, p. 27). The commander’s aim describes a summary of the orders given, including a mutually desired end state that the crew must strive for; for example, what will be done, against what and whom and how it will be done (Swedish Armed Forces 2018, p. 22). Between each scenario during the day, the training was stopped for a couple of minutes to give the participants time to fill in the questionnaires before the scenario continued. Information about the quick stops was given verbally during the Navy ETS scenario and through the internal speaker system onboard Vulcanus II. Before the day ended, the whole crew gathered for a summary of the day.

### Analysis

The completed questionnaires were analysed using SPSS version 29 for Windows. Wilcoxon signed rank test was used to make comparisons between the low- and high-fidelity as well as between part 1 and 2 in the scenario. NASA-TLX total score was computed using the mean of the six dimensions for each time they were rated by each participant. Data from the last escalating part (part 3) was omitted because some participants had not responded by the end of the day.

### Ethical approval

This research complied with the tenets of the Declaration of Helsinki and was approved by the Ethical Review Board in Linköping (Dnr 2013/163-31. Informed consent was obtained from each participant. The participants were informed that their participation was voluntary and that they could withdraw from the study at any time without explanation and without impact on their current duty or education in progress.

## Results

**Fig. 9:**
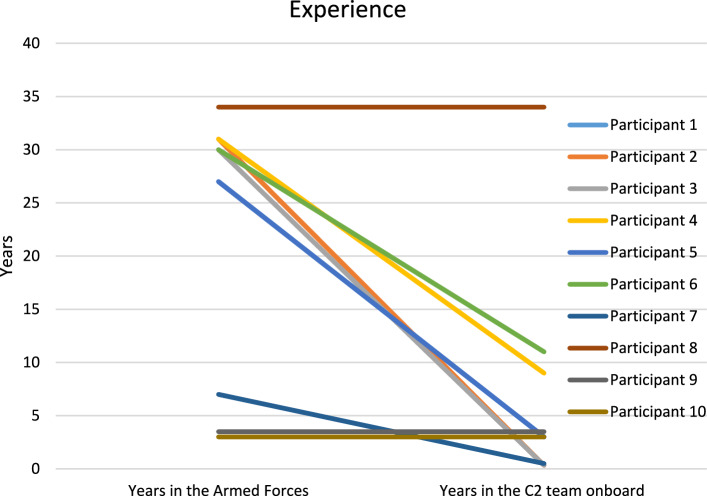
Years in the Swedish Armed Forces and years in the role onboard

The participants ranged in age from 23 to 57 years (mean 39.8 years) and consisted of 9 men and 1 woman. As seen in Fig. 9 all participants had varying experience in the Swedish Armed Forces (mean, 21.7 years; range, 3–34 years) and varying experience in their position onboard the vessel (mean, 6.8 years; range, 4 months to 34 years). The results showed no significant differences in workload regarding the NASA-TLX rating between the simulations. When comparing the results from Grier (2015), the findings are in line with the global TLX data values for C^2^ teams.

Experiences are presented in Fig. 9. Years in the Swedish Armed Forces in total and years in the C^2^ team onboard.

The descriptive statistics for the variables for ETS and Vulcanus II are presented in Table 1.

**Table 1:** Dependent variables for ETS and Vulcanus II in the first and second part of the scenario

Dependent measure	ETS		Vulcanus II	
	Part 1	Part 2	Part 1	Part 2
Mental demand Physical demand Temporal demand Performance Effort Frustration NASA-TLX total	64.8 (25.85) 9.6 (17.41) 43.8 (18.56) 62.7 (28.04) 45.7 (22.36) 27.9 (28.71) 42.5 (13.73)	70.1 (21.92) 13:9 (30.33) 53.4 (24.59) 70.3 (32.67) 47.4 (33.78) 36.2 (26.41) 48.6 (14.32)	56.7 (20.70) 20.6 (33.28) 43.3 (20.22) 74.5 (32.48) 53.9 (24.14) 38.9 (36.47) 48,1 (13.08)	58.5 (29.13) 19.5 (30.02) 48.7 (25.79) 71.8 (32.17) 51.8 (33.54) 47.3 (33.70) 48.5 (17.43)
Situation awareness	78.3 (27.73)	84.4 (18.90)	69.5 (32.96)	86.6 (14.94)
Psychological combat readiness	99.1 (17.47)	95.3 (17.70)	103.1 (8.37)	104.0 (10.36)
Physical combat readiness	105.3 (10.05)	93.6 (34.80)	105.3 (8.09)	105.8 (8.66)
Combat readiness of the ship	63.7 (26.73)	49.2 (18.67)	83.9 (16.93)	78.7 (19.96)

Mean and standard deviation (SD) in parentheses

The dependent measures were analysed using Wilcoxon signed rank test, presented in Table 2.

**Table 2:** Summary of comparisons

Measures	N	Mean diff	CI	Z-value	*p*-value
Mental demand 1 low versus high	10	8.1	− 11.7; 27.9	− 1.376	0.169
Mental demand 2 low versus high	9	10.8	0.91; 20.7	− 2.1	0.036*
Physical demand 1 low versus high	10	− 11.0	− 24.9; 2.9	− 1.380	0.168
Physical demand 2 low versus high	10	− 5.6	− 11.7; 0.53	− 1.893	0.058
Temporal demand 1 low versus high	10	0.50	− 15.0; 16.0	− 0.255	0.799
Temporal demand 2 low versus high	10	4.75	− 14.0; 23.4	− 0.510	0.610
Performance 1 low versus high	10	− 11.8	− 40.6; 17.0	− 0.889	0.374
Performance 2 low versus high	10	− 1.5	− 33.6; 30.5	− 0.357	0.721
Effort 1 low versus high	10	− 8.2	− 34.6; 18.2	− 1.070	0.285
Effort 2 low versus high	10	− 4.3	− 14.3; 5.6	− 1.071	0.284
Frustration 1 low versus high	9	− 6.9	− 44.6; 30.8	0.000	1.0
Frustration 2 low versus high	10	− 11.1	− 27.2; 5.0	− 1.070	0.285
NASA-TLX total 1 low versus high	9	− 4.19	− 17.28; 8.91	− 0.889	0.375
NASA-TLX total 2 low versus high	9	− 1.34	− 8.60; 5.92	− 0.420	0.674
SA 1 low versus high	10	8.8	− 20.3; 38.0	− 0.561	0.575
SA 2 low versus high	10	− 2.1	− 13.0; 8.7	− 0.415	0.678
Psychological combat readiness 1 low versus high	10	− 3.9	− 16.4; 8.5	− 0.210	0.833
Psychological combat readiness 2 low versus high	10	− 8.6	− 22.2; 4.9	− 1.120	0.263
Physical combat readiness 1 low versus high	10	0.00	− 2.7; 2.7	− 0.318	0.750
Physical combat readiness 2 low versus high	10	− 12.2	− 39.8; 15.4	− 0.178	0.859
Combat readiness of the ship 1 low versus high	10	− 20.2	− 36.5;− 4.0	− 2.091	0.037*
Combat readiness of the ship 2 low versus high	9	− 27.1	− 46.9; − 7.4	− 2.201	0.028*

The table shows number of cases, mean differences based on t-tests, confidence interval based on t-tests, Z-values based on Wilcoxon, and *p*-values based on Wilcoxon

Low versus high refers the two simulation exercise settings: ETS (low) and Vulcanus II (high)

The number 1 and 2 refers to the consecutive escalating parts of the scenario

Measures in TLX-scale 0–100

*Describes a *p* value < 0.05

The dependent measure, combat readiness of the ship, yielded significant results. That is, a difference was detected between the two simulation environments (ETS vs Vulcanus II) as well as between the consecutive escalating parts (parts 1 and 2) of the scenario. The participants achieved a higher level of combat readiness of the ship in the high-fidelity setting. A difference was found between the first and the second part of the scenario in the high-fidelity setting where a higher level of combat readiness was achieved in the first part than in the second part. Furthermore, mental demand in the second part of the scenario showed significant result. The participants reached a higher level of mental demand in the low-fidelity setting. No other statistically significant differences were found.

## Discussion

The research compared two simulation environments to evaluate the performance of a Swedish naval C^2^ team using indirect measures such as workload, combat readiness, and SA.

The first question, addressing whether low-fidelity simulation settings differed from high-fidelity settings in experienced workload using NASA-TLX, revealed no significant differences, a noteworthy finding. Participant experience and commitment may explain the non-significance, although the small sample size does not rule out potential differences, prompting further consideration of how SBT is conducted and at what cost. The matter of costs and extensive resources required in high-fidelity simulations are in line with previous research (Fragapane et al. 2018; Massoth et al. 2019; Norman et al. 2012). In other research, the NASA-TLX was proved to be sensitive to experience (Barajas-Bustillos et al 2023) which may have had an influence on the results in this study as well. In general, the members had worked in the Armed Forces for many years before being selected to participate in the C^2^ team.

The second question, investigating differences in perceived combat readiness between low- and high-fidelity settings, identified a significant decrease in ship combat readiness between scenario parts 1 and 2 in both settings. The scenarios’ intentional design, where the second part was perceived as more challenging, could explain this. However, in the high-fidelity setting (Vulcanus II), participants exhibited increased performance despite being under a red (highest) threat level, necessitating further research to understand why combat readiness was considered higher in this training facility. The results indicate that in the complex high-fidelity setting, participants perceived the environment as more realistic and having a closer resemblance to the actual onboard vessel environment, aligning with previous research findings (Dieckman et al. 2007; Banks 2010; Tusher et al. 2023).

The third question, exploring differences in SA between low- and high-fidelity settings, yielded no significant results. According to Endsley (1995a), three levels of SA are part of the theoretical framework of SA. Questions arose about the participants’ SA levels during different scenario parts, given the potential influence of the simulation environment on their perceptions. Even though the participants had previous experience of internal battle, the simulation environment and scenarios may have affected how they perceived the SA. The different levels of SA include how the status and dynamics of relevant elements in the environment are perceived, for example, multiple situational elements such as systems or environmental factors, and processes of pattern recognition (Endsley 1995b). The study focused on the SBT itself rather than the vessel’s onboard capability, but the issue of SA requires additional investigation.

The results showed that the low-fidelity setting was perceived as more mentally demanding than the high-fidelity setting. This may be due to the lower level of information in the low-fidelity setting, usually received in the actual environment during ongoing events. Another cause can be that the participants needed more effort to engage in the different parts of the scenario—which in turn may have influenced the increased level of mental demand in the low-fidelity setting. When performing the same scenario in the high-fedelity setting, the participants knew what to expect and they had knowledge of the different parts of the scenario which can explain the lower level of mental demand.

In summary, this study showed no significant differences in workload and SA or between the low- and high-fidelity settings. This is in line with other research such as the systematic review and meta-analysis by Osborne et al. (2022) where no significant difference was found in learners’ knowledge or skills when comparing high-fidelity simulation with low-fidelity simulation. The only significant differences in this study was the combat readiness of the ship and mental demand.

This study enhances understanding of how Swedish naval C^2^ teams can be trained. It suggests that Navy ETS, a form of TTX, can be employed for low-fidelity SBT, offering training in a simpler, yet challenging environment with effects comparable to high-fidelity settings. The SBT took place in a naval context with the same escalating scenario carried out in low- and high-fidelity simulation settings, characterized by working under time pressure, stress, workload and conceivably with goals for the tasks given, which may provide generalizability based on our findings (Klein et al. 1993).

To be able to perform command and control on a ship effectively in the context of naval operations, personnel must be capable of efficiently utilizing available resources and aligning their actions with the commander’s aim. This requires comprehensive training. Naval C^2^ team training typically occurs in a high-fidelity setting, requiring much time and incurring substantial costs due to the requisite allocation of material and personnel resources. As seen in this study, low-fidelity simulations demand fewer resources, thereby presenting a more effective alternative concerning time and money. The working conditions outlined in this study may be relevant to other professions and other domains than those who will serve on naval warships. Low-fidelity simulations like ETS or other TTX can be adapted for various contexts and teams, for example in hospital preparedness (Söderin et al. 2023), in managing future pandemic outbreaks (Mishra et al., 2023), in flood risk management (Shen and Jiang 2023), in disaster preparedness (Mahdi et al. 2023) or in crisis management (Buhagiar and Anand 2023). As described in the introduction, performance framework, fidelity as well as workload, combat readiness and SA are important concepts in simulations. These concepts can also be used in training of first responders (Police officers, Firefighters, and Emergency Medical Services [EMS]). Research in medical services show that performance assessments in simulated scenarios closely reflect behaviors in real disaster situations. Measurable factors like accuracy, timing, and the sequence of actions can be utilized to evaluate performance (Baetzner et al. 2022). In this study was the NASA-TLX used to examine performance which can be applied in training of first responders. Other tools to support in training of first responders are virtual reality [VR] (Wilkerson et al. 2008; Haskins et al. 2020), and extended reality (XR), which encompasses virtual, augmented and mixed reality [VR, AR, and MR] (Otero-Varela 2023).

Low-fidelity analogue simulations support TTX by providing the participants with an advanced starting point for upcoming simulations, develop their ability to take action and provide training in communication and organizational matters and increase their understanding for methodology and the chain of command. While low-fidelity simulations may pose challenges for novices due to their lower realism, experienced crew members can grasp the seriousness of the scenarios. In a study by Pettersson et al. (2021), discussions were raised concerning the participants’ behaviour in a simulated event, and the risk of the behaviour not being in line with actions taken in an actual event. The study’s participants, having operational unit service experience, likely mirrored realistic behaviour, aligning with the principle of “train as you fight” (NATO Standardization Office 2019).

Concerning the questionnaires used in this study, the participants were already familiar with the method of using questionnaires and required only a brief explanation. The method's advantages included uniformity in providing the same questions to all participants simultaneously, without any influence from researchers. The surveys and questionnaires proved to be time-efficient in the course of this research. Another strength of the study was the repetition of the same scenario in both low- and high-fidelity simulation settings within an unique and controlled environment.

Training and combat readiness are crucial for mission completion (Naval Surface Force United States Pacific Fleet 2012) as emphasized by Rear Admiral Ewa Skoog Hasslum, Chief of the Swedish Navy, who states “It is extremely important for us to train, we must train a lot more than we have done previously” (Swedish Armed Forces, third naval flotilla 2021) (quote translated by the first author). The fact that we need to train is clear.

### Limitations of the study

There were drawbacks to consider. The method did not allow for further elaboration of answers or follow-up questions. The NASA-TLX questionnaire was unfamiliar to the Swedish naval C^2^ team, as participants typically do not preview questionnaires before a study. The measurement employed in this study might be perceived as intrusive during the exercise, potentially impacting the results. Questionnaires disrupting the exercise could heighten participants' frustration, negatively influencing the parameters of certain ratings. Although NASA-TLX was chosen for its quick completion, it might have disrupted the high-fidelity simulation setting, explaining the missing data in the last escalating part of the scenario (part 3), which was excluded from the research analysis to maintain accuracy. Nevertheless, this research suggests that using NASA-TLX subscales is a valuable tool for assessing workload in Swedish naval C^2^ teams during simulations.

Another limitation was the low number of participants, and may not reflect other Swedish naval C^2^ teams. Additional research on an increased number of participants belonging to other C^2^ teams should strenghten the results. Due to this limitation, it is difficult to generalize the results. Increasing the number of participants would allow more precise comparisons to be made between the naval C^2^ team and the other crew during SBT to examine workload, combat readiness and SA.

### Future research

The inquiry into learning can be explored in the context of SBT to determine whether a low or high workload holds greater significance for the learning process. Investigating participants’ learning when engaged in SBT is a compelling avenue, and concurrently assessing instructors’ learning during the same exercise adds an extra layer of significance. This line of inquiry extends beyond SBT and could prove crucial in various domains, such as crisis management. This study also underscores the need to improve knowledge of the type of SBT that is preferable, making informed choices based on factors like cost benefits and resource availability.

### Practical implications

Theoretical knowledge in the low-fidelity setting seems to connect with real-world application in the high-fidelity setting. Results indicate that the combination of settings as in this study prepare the C^2^ team to handle complex tasks. The practical implications arising from this research indicate that integrating regular SBT sessions in preparation for maritime operations can increase crew preparedness. Maritime organizations should consider incorporating low-fidelity simulation settings into their standard procedures to effectively support the training of naval C^2^ teams. Low-fidelity simulations, conducted indoors with fewer instructors and reduced equipment, offer a more effective approach in both time and costs, allowing for an increased number of training sessions. In contrast to large-scale scenarios which demand substantial resources, including personnel, materials, and diversely challenging environments, the cost-effectiveness of implementing low-fidelity scenarios, as demonstrated in this study, should be embraced. This is in line with a study by Ritter et al (2023) where the low-fidelity simulation showed reduced time expenditure with minimal delays, and the high-fidelity simulation basically took as much time as the real-world task. Massoth et al (2019) alleges that the use of high-fidelity simulation can lead to inefficient use of valuable resources and an imbalanced cost–benefit ratio. In the military, simulators have been used for cost-effective reasons as well as for realistic training for many years (Waldenström 2012). Furthermore, policymakers could use this research to update MET and SBT for individuals to enhance a safer and more resilient maritime sector.

### Conclusions

The study suggests that low-fidelity simulation settings for internal battles are comparable to high-fidelity simulation settings for Swedish naval C^2^ teams on board. The work emphasizes its relevance to Naval research in preparing units for various situations they might face. Comparison of the two simulation environments for training of naval C^2^ teams showed no significant differences except for the combat readiness of the ship. Therefore, the study suggests that the benefits of low-fidelity simulation settings for internal battles are comparable to those of high-fidelity simulation settings for Swedish naval C^2^ teams on board. Low-fidelity SBT can be conducted more frequently, and the dynamics of low-fidelity simulations can be easily adjusted. The efficiency in low-fidelity settings regarding time, costs and resources ought to be of interest for both the Armed Forces and other organizations. Administering SBT before actual combat may facilitate the adaptation to new challenges, aligning with the evolving nature of the future battlefield (battlespace).

## Declarations

### Human or animal participants

This article does not contain any studies with animals performed by any of the authors.
